# In Vitro Activity of Aztreonam–Avibactam, Cefiderocol, and Conventional Antimicrobials Against Metallo-β-Lactamase-Producing Gram-Negative Urinary Isolates: A Systematic Review and Local Single-Center Retrospective Evaluation in a Tertiary Care Hospital (2016–2025) and Evaluation of Novel Agents (2021–2025)

**DOI:** 10.3390/antibiotics15101010

**Published:** 2026-10-11

**Authors:** Lucía Requena-Fernández, Noemí Camacho-Blanco, Antonio Rosales-Castillo, Leticia Castellano-Sánchez, José Gutiérrez-Fernández

**Affiliations:** 1 Department of Microbiology, School of Medicine, University of Granada- Instituto de Investigación Biosanitaria de Granada,18014 Granada, Spain; 2 Clinical Medicine and Public Health Doctoral Program, PostGraduate School, University of Granada, 18012 Granada, Spain; 3 Department of Internal Medicine, Hospital Universitario Virgen de las Nieves- Instituto de Investigación Biosanitaria de Granada, 18012 Granada, Spain; 4 Microbiology Laboratory, Hospital Universitario Virgen de las Nieves- Instituto de Investigación Biosanitaria de Granada, 18012 Granada, Spain

**Keywords:** metallo-β-lactamase, Gram-negative bacteria, cefiderocol, aztreonam–avibactam, antimicrobial susceptibility, Enterobacterales, *P. aeruginosa*, carbapenem resistance

## Abstract

**Background:** Metallo-β-lactamase (MBL)-producing Gram-negative bacteria represent one of the greatest therapeutic challenges in contemporary clinical microbiology because of their extensive antimicrobial resistance and the limited availability of effective treatment options. Aztreonam–avibactam and cefiderocol have emerged as promising therapeutic alternatives; however, comparative evidence from routine clinical practice remains limited. **Methods:** A systematic literature search was conducted in PubMed to identify studies published between 2015 and 2025 evaluating the in vitro activity of aztreonam–avibactam and cefiderocol against MBL-producing Gram-negative bacteria. A total of 22 fully accessible studies meeting the eligibility criteria were included and analyzed in the systematic review. In parallel, a retrospective cross-sectional study was performed including all urinary isolates producing MBL recovered at the Microbiology Laboratory of Virgen de las Nieves University Hospital (Granada, Spain) between January 2016 and December 2025. Antimicrobial susceptibility profiles for both novel agents and conventional antibiotics were analyzed. **Results:** Twenty-three studies met the inclusion criteria for the systematic review, of which 22 fully accessible articles were analyzed. In the systematic review, Enterobacterales accounted for 70.0% of the reported MBL isolates, with New Delhi metallo-β-lactamase (NDM) being the predominant enzyme globally (50.5%), followed by Verona integron-encoded metallo-β-lactamase (VIM) (39.4%). The crude aggregated *in vitro* susceptibility proportion across the literature was markedly higher for aztreonam–avibactam than for cefiderocol (93.1% vs. 58.1%). Conversely, in our local urinary cohort (*n* = 182), Enterobacterales were also the predominant pathogens (51.6%), but VIM was the most frequently detected carbapenemase (*n* = 107). In this local cohort, aztreonam–avibactam demonstrated 100% in vitro susceptibility against Enterobacterales (54/54) and exhibited a 77.8% wild-type (WT) phenotype against *Pseudomonas* spp. (35/45). Cefiderocol exhibited excellent activity against *Pseudomonas* spp. (86.2%, 50/58) but significantly lower susceptibility rates among Enterobacterales (39.6%, 21/53). Among conventional antimicrobials, in vitro susceptibility in Enterobacterales to gentamicin decreased significantly over time (from 62.5% to 32.8%; FDR-adjusted *p* = 0.029), whereas amikacin in vitro susceptibility significantly improved (from 60.0% to 88.1%; FDR-adjusted *p* = 0.048). Colistin maintained high susceptibility rates, ranging from 87.8% to 100% in Enterobacterales and 77.8% to 88.2% in *Pseudomonas* spp. Agents such as nitrofurantoin (restricted exclusively to *Escherichia coli*) and trimethoprim–sulfamethoxazole or fosfomycin were not evaluated for *Pseudomonas* spp., due to intrinsic resistance or absence of established EUCAST clinical breakpoints. **Conclusions:** Aztreonam–avibactam and cefiderocol exhibit promising but highly species-specific in vitro activity against MBL-producing Gram-negative bacteria. While colistin and amikacin remain active conventional in vitro alternatives, these laboratory findings represent potential microbiological potency rather than guaranteed clinical success. Prospective clinical trials and real-world outcome studies remain essential to evaluate the clinical efficacy, patient outcomes, and comparative in vivo effectiveness of these regimens in daily practice.

## 1. Introduction

The global dissemination of multidrug-resistant Gram-negative bacteria has become one of the most pressing public health threats, accounting for more than 35,000 deaths annually across the European Union, Iceland, and Norway [1]. Current projections suggest that antimicrobial resistance could become the leading cause of death worldwide by 2050 if effective containment strategies are not implemented [2].

Carbapenemase-producing Gram-negative bacilli exhibit extensive resistance, limiting the number of active agents available in vitro, prolonged hospitalization, and increased mortality [3]. Specifically, invasive infections caused by MBL-producing strains, such as bloodstream infections and nosocomial pneumonias, carry exceedingly high mortality rates, typically ranging between 30% and 50% in European settings [1,3]. This high lethality is particularly pronounced when initial empirical treatment is inadequate or delayed [3].

Within the intensive care unit, these infections represent a major challenge and are strongly driven by invasive procedures, mechanical ventilation, and indwelling devices, which facilitate colonization and prolong hospital stays [3]. However, there is a worrying and progressive shift in their epidemiology, with community-onset presentations—most notably complicated urinary tract infections presenting to emergency departments—rising globally [1]. This community spread severely complicates empirical antibiotic selection, as clinicians face multidrug-resistant phenotypes in patients without classic nosocomial risk factors [1,3].

Reflecting their increasing global clinical importance, carbapenem-resistant Enterobacterales remain classified as critical-priority pathogens in the latest World Health Organization (WHO) bacterial priority pathogen list, whereas *P. aeruginosa* has been reclassified as a high-priority pathogen, highlighting the urgent need for novel therapeutic strategies [4].

MBLs, classified as Ambler class B enzymes, frequently coexist with class A and class C serine β-lactamases, including extended-spectrum β-lactamases (ESBLs) and AmpC enzymes [5]. Although MBLs hydrolyze nearly all β-lactam antibiotics, monobactams such as aztreonam remain intrinsically stable to MBL-mediated hydrolysis. However, the frequent coproduction of ESBLs, AmpC enzymes, or serine carbapenemases such as KPC compromises aztreonam activity, substantially limiting its clinical usefulness when administered alone [6].

Avibactam is a non-β-lactam β-lactamase inhibitor with potent activity against Ambler class A, class C, and selected class D enzymes [7,8]. By inhibiting accompanying serine β-lactamases, avibactam restores the antibacterial activity of aztreonam against MBL-producing organisms, making the aztreonam–avibactam combination a promising in vitro therapeutic candidate [9,10].

Alongside aztreonam–avibactam, cefiderocol has emerged as another promising option for the treatment of multidrug-resistant Gram-negative infections. This novel siderophore cephalosporin exploits bacterial iron transport systems to achieve enhanced intracellular penetration while maintaining remarkable stability against hydrolysis by most β-lactamases. Consequently, cefiderocol exhibits potent activity against many carbapenem-resistant Gram-negative pathogens, including MBL-producing isolates [11].

Despite the growing clinical interest in these agents, comparative data evaluating their in vitro activity against MBL-producing bacteria remain limited, particularly at the local epidemiological level. Since antimicrobial susceptibility patterns may vary considerably according to the regional distribution of carbapenemase genes, local surveillance studies are essential to optimize empirical and targeted antimicrobial therapy.

Accordingly, the present study aimed to evaluate the in vitro activity of cefiderocol and aztreonam–avibactam against MBL-producing Gram-negative bacteria by combining a systematic review of the international literature (2015–2025) with a ten-year susceptibility analysis of urinary isolates recovered at Virgen de las Nieves University Hospital (Granada, Spain). In addition, the activity of conventional antimicrobials was assessed to identify older agents retaining susceptibility against MBL-producing isolates.

## 2. Materials and Methods

### 2.1. Systematic Review

#### 2.1.1. Protocol Registration and Compliance

**Figure 1:**
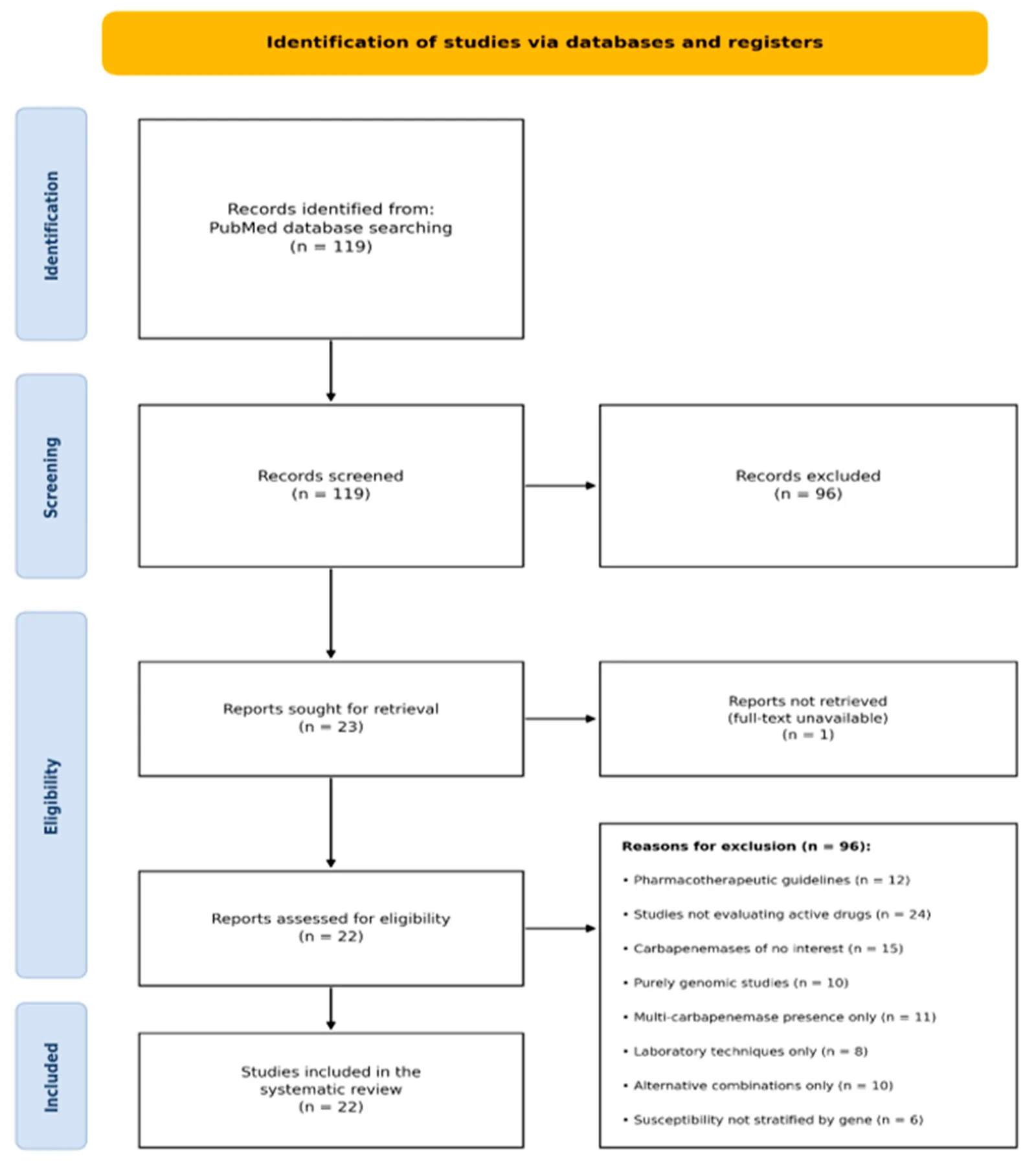
PRISMA 2020 flow diagram of the study selection process for the systematic reviewPRISMA: Preferred Reporting Items for Systematic Reviews and Meta-Analyses.

This systematic review was conducted in strict accordance with the Preferred Reporting Items for Systematic Reviews and Meta-Analyses (PRISMA) 2020 guidelines, as illustrated in the selection flow (Figure 1). The review protocol was prospectively registered in the International Prospective Register of Systematic Reviews (PROSPERO) on 5 February 2026 (registration number: CRD420261298047).

#### 2.1.2. Information Sources and Search Strategy

A comprehensive, systematic literature search was performed in PubMed/MEDLINE, selected as the primary benchmark database in biomedical and clinical microbiology research, covering publications through 5 February 2026 to identify studies evaluating the in vitro activity of cefiderocol and aztreonam–avibactam against MBL-producing Gram-negative bacteria. Study eligibility was restricted to articles published in English or Spanish.

The search strategy incorporated a combination of Medical Subject Headings (MeSH), Title/Abstract field tags ([TiAb]), broad taxonomical categories to avoid omitting relevant MBL-producing species, and explicit publication date and language limits.

The complete, executable Boolean query applied in PubMed/MEDLINE was as follows:

(("Metallo-beta-Lactamases"[Mesh] OR "metallo-beta-lactamase"[TiAb] OR "MBL"[TiAb] OR "VIM"[TiAb] OR "NDM"[TiAb] OR "IMP"[TiAb]) AND ("cefiderocol"[TiAb] OR "aztreonam-avibactam"[TiAb] OR "aztreonam/avibactam"[TiAb]) AND ("Gram-Negative Bacteria"[Mesh] OR "Gram-negative"[TiAb] OR "Enterobacterales"[TiAb] OR "Pseudomonas aeruginosa"[TiAb] OR "*Klebsiella pneumoniae*"[TiAb] OR "*Escherichia coli*"[TiAb])) AND (2015/01/01:2026/02/05[PDAT]) AND (english[lang] OR spanish[lang])

This search strategy yielded a total of 119 unique records.

#### 2.1.3. Study Selection and Reviewer Procedures

The comprehensive literature search in PubMed/MEDLINE retrieved a total of 119 unique records. Since PubMed served as the single benchmark database, no inter-database deduplication step was required, and all 119 identified records proceeded directly to screening.

Study eligibility was evaluated using a two-stage process. First, two reviewers (L.R.-F. and N.C.-B.) independently and in duplicate screened the titles and abstracts of all 119 unique records against pre-specified eligibility criteria. A total of 96 records were excluded for specific reasons: pharmacotherapeutic guidelines (*n* = 12), studies not evaluating target active agents (*n* = 24), carbapenemases of no interest (*n* = 15), multi-carbapenemase presence only (*n* = 11), purely genomic studies (*n* = 10), alternative combinations only (*n* = 10), laboratory techniques only (*n* = 8), and susceptibility not stratified by resistance gene (*n* = 6).

Second, 23 reports were sought for full-text retrieval. One report was unavailable despite extensive institutional library retrieval efforts. The remaining 22 full-text articles were retrieved, assessed for eligibility, and included in the final qualitative synthesis. Any potential discrepancies during screening or eligibility assessment were resolved through consensus or consultation with a senior third reviewer (L.C.-S.).

#### 2.1.4. Data Extraction and Data Items

Data from the 22 fully accessible included studies were extracted independently and in duplicate by two reviewers (L.R.-F. and N.C.-B.) using a standardized Microsoft Excel template. The extracted data items included: first author, publication year, study country, bacterial species or group evaluated, underlying MBL genotype (e.g., *bla*_VIM_, *bla*_NDM_, *bla*_IMP_), sample size (*n*), susceptibility testing methodology (gradient diffusion, broth microdilution, or ceftazidime–avibactam/aztreonam disk synergy), susceptibility percentages (%S), and minimum inhibitory concentration (MIC) ranges, MIC_50_, and MIC_90_ values. For synergy assays, the visual demonstration of synergistic activity was accepted to classify an isolate as susceptible, even in the absence of a quantitative MIC value.

#### 2.1.5. Methodological Quality and Risk of Bias Assessment

The methodological quality and risk of bias of the eligible studies were evaluated independently in duplicate by two authors (L.R.-F. and N.C.-B.). Because the included papers consist of preclinical, in vitro microbiological susceptibility investigations, standard clinical assessment tools (such as the Cochrane Risk of Bias tool or the Newcastle–Ottawa Scale) are not directly applicable. Therefore, we adapted the Joanna Briggs Institute (JBI) Critical Appraisal Tool for Prevalence Studies. The quality appraisal was performed on the 22 fully accessible studies (excluding one eligible study due to full-text unavailability) and focused on four critical methodological domains essential to minimize bias in clinical microbiology research:

- Isolate Selection: Evaluation of whether isolates represented a well-defined, non-duplicate clinical cohort (Low Risk) vs. convenience or duplicate strains (High/Unclear Risk).
- AST Standardization: Adherence to standardized reference methods (such as broth microdilution or agar dilution) and recognized consensus guidelines (Low Risk).
- Breakpoint Criteria: Explicit use of up-to-date interpretive reference guidelines (such as EUCAST or CLSI) (Low Risk). Studies relying on qualitative synergy assays (e.g., disc-stacking or strip placement without formal MIC clinical breakpoints) were assigned a Moderate Risk of Bias for this domain.
- Molecular Confirmation: Use of validated molecular techniques (such as PCR or sequencing) to confirm underlying carbapenemase genotypes (Low Risk).

A complete study-level evaluation matrix detailing domain judgments across all 22 included studies is provided in Supplementary Material S1 (Table S1).

#### 2.1.6. Synthesis Methods and Reporting Bias Evaluation

Susceptibility rates and MIC distributions extracted from the literature are summarized using descriptive statistics. Because the included studies reported raw in vitro susceptibility proportions (descriptive microbiological parameters) across highly heterogeneous, non-comparative preclinical cohorts, rather than comparative intervention effects (such as odds ratios or relative risks), quantitative meta-analysis, forest plots, and formal statistical publication bias evaluations (such as funnel plots or Egger’s test) were not mathematically applicable. Instead, reporting and publication bias were qualitatively addressed by: (i) conducting a comprehensive search in PubMed/MEDLINE; (ii) manually searching reference lists; and (iii) verifying that all included studies reported susceptibility rates for both active and control agents across all tested species to prevent selective reporting.

### 2.2. Local Susceptibility Study

A retrospective, cross-sectional descriptive study was conducted using microbiological data generated by the Microbiology Laboratory of Virgen de las Nieves University Hospital (HUVN), Granada, southern Spain. The Hospital is a regional healthcare complex made up of three centers (General Hospital of Specialties, Maternal-Child Hospital, and Hospital of Neuro-Traumatology and Rehabilitation) that provides third-level care activity in the province. In our study, the assisted population consisted primarily of patients from specialized care, reflecting their high clinical and microbiological complexity. The Microbiology Department operates under a quality management system accredited by the Spanish National Accreditation Body (ENAC) [12], which guarantees the validity and reliability of our results.

An anonymized database including all clinical urine cultures requested because of suspected urinary tract infection (UTI) between 1 January 2016 and 31 December 2025 was reviewed. No clinical exclusion criteria were applied; however, there was a lack of explicit information on clinical urinary symptoms in the electronic records. Urine specimens included midstream urine, urine collected through permanent or temporary urinary catheters, pediatric urine collection bags, and nephrostomy catheter samples.

Over this 10-year study timeline (2016–2025), a sequential diagnostic screening and selection flow was applied. Out of approximately 139,570 total requested urine cultures reviewed, 48,072 specimens displayed significant bacteriuria (overall 34.4% positivity rate), from which 36,054 Gram-negative bacilli isolates were recovered. Initial carbapenemase screening identified ~920 carbapenem-non-susceptible isolates, and ~400 isolates underwent confirmatory testing. This confirmed 205 MBL-producing isolates (76 in 2016–2023 and 129 in 2024–2025). Following strict epidemiological deduplication, a final cohort of 182 non-duplicate MBL-producing clinical isolates was included for definitive susceptibility analysis.

Isolate eligibility was strictly restricted to episodes presenting significant bacteriuria, defined as a uropathogen growth ≥10^5^ CFU/mL for conventional specimens (midstream urine or pediatric collection bags) or ≥10^4^ CFU/mL for catheter-derived (temporary or permanent) and nephrostomy specimens. Clinical episodes were categorized strictly according to the requesting healthcare setting as recorded in the laboratory information system, rather than inferring definitive epidemiological acquisition mechanisms. Specifically, cultures were classified into two care settings: Emergency Department and Outpatient setting (cultures requested by primary care centers, outpatient clinics, or emergency departments) versus Inpatient Ward setting (cultures requested by inpatient hospital medical or surgical units, or intensive care units). The 10-year study timeline was stratified into two distinct five-year periods—the baseline period (2016–2020) and the recent period (2021–2025)—based on three key diagnostic, therapeutic, and epidemiological milestones that occurred in 2021. Chronologically, 2021 marked the systematic clinical implementation of the NG-Test^®^ Carba 5 lateral flow immunochromatographic assay in our laboratory routine, enabling immediate prospective real-time identification of carbapenemase families instead of the retrospective phenotypic/molecular screening methods used during the first period (2016–2020). Clinically, 2021 also marked the routine clinical availability and laboratory susceptibility evaluation of the novel agents cefiderocol and aztreonam–avibactam at our tertiary care hospital. Epidemiologically, the year 2021 represented a major inflection point in our geographical area; the severe clinical pressure on intensive care units during the COVID-19 pandemic triggered a dramatic surge in infection and colonization rates by carbapenem-resistant and MBL-producing uropathogens (particularly VIM-producing *P. aeruginosa* and NDM-producing Enterobacterales) due to intense selection pressure, as previously described in our setting. Consequently, the 2016–2020 period represents a pre-pandemic baseline of low endemicity, while the 2021–2025 period represents a high-endemic pandemic/post-pandemic era under novel diagnostic and therapeutic algorithms.

To isolate the target population, a standardized diagnostic screening and confirmation flow was applied to all processed specimens. Gram-negative bacilli recovered from clinically significant urine cultures underwent species identification via MALDI-TOF MS (Bruker Daltonik GmbH, Bremen, Germany) and automated susceptibility testing using the MicroScan^®^ WalkAway or VITEK^®^ 2 systems. Isolate screening for carbapenemase production was triggered by carbapenem non-susceptibility according to EUCAST criteria (specifically, an ertapenem MIC > 0.5 mg/L and/or a meropenem/imipenem MIC > 0.12 mg/L) [13]. All flagged non-susceptible isolates were subsequently confirmed using the NG-Test^®^ Carba 5 immunochromatographic assay to identify KPC, OXA-48, NDM, VIM, and IMP carbapenemase families. Only those isolates confirmed to produce at least one MBL genotype (VIM, NDM, or IMP), either as single producers or co-producing serine carbapenemases (such as OXA-48), were included in the final analytical cohort, yielding a final sample size of 182 non-duplicate MBL-producing clinical isolates.

### 2.3. Microbiological Procedures

Urine specimens obtained from patients with suspected UTI were collected according to routine clinical practice using midstream urine collection, temporary catheterization, permanent urinary catheters, or pediatric urine collection bags to minimize contamination.

All specimens were processed according to standardized laboratory procedures accredited by the Spanish National Accreditation Body (ENAC) [12]. Samples were received either in sterile containers or in boric acid preservation tubes (Vacutainer^®^, Becton Dickinson, Franklin Lakes, NJ, USA).

Quantitative urine cultures were performed on CHROMID^®^ CPS Elite chromogenic agar (bioMérieux, Marcy-l’Étoile, France) and incubated at 37 °C for 24–48 h. For samples from the Nephrology Department, Columbia sheep blood agar (bioMérieux, France) incubated under a CO_2_ atmosphere was additionally used.

Clinically significant cultures were defined as ≥10^5^ CFU/mL for one or two uropathogens in conventional urine specimens, ≥10^4^ CFU/mL in urine obtained by temporary catheterization, or 10^4^–10^5^ CFU/mL for a single uropathogen (10^3^–10^4^ CFU/mL in temporary catheter specimens).

Microorganism identification was performed using matrix-assisted laser desorption/ionization time-of-flight mass spectrometry (MALDI-TOF MS, Bruker Daltonik GmbH, Bremen, Germany) and/or the MicroScan^®^ WalkAway system (Beckman Coulter, Brea, CA, USA). Antimicrobial susceptibility testing (AST) was carried out using a sequential laboratory workflow that evolved chronologically over the 10-year study period. Between 2016 and 2020, automated AST was performed using the MicroScan^®^ WalkAway (Beckman Coulter) system with dMLite, NM45, or NM46 commercial panels, occasionally supplemented by manual gradient diffusion (Etest^®^, bioMérieux, France) for specific confirmatory testing. From 2021 through 2025, the VITEK^®^ 2 automated system (bioMérieux, France) utilizing AST-N377 or AST-N399 cards was integrated as an additional AST platform alongside the MicroScan^®^ WalkAway. To maintain strict temporal comparability and eliminate potential bias arising from changes in susceptibility platforms, panels, or historical interpretive guidelines over time, the qualitative susceptibility categories (S/I/R) recorded at the time of isolation were discarded. Instead, raw MIC values for all conventional antimicrobials were extracted directly from the MODULAB^®^ laboratory database (Werfen, Barcelona, Spain).

For isolates displaying carbapenem MIC values above the EUCAST clinical breakpoints for carbapenemase screening [13], carbapenemases were first screened using the colorimetric Neo-Rapid CARB Kit (Rosco Diagnostic A/S, Taastrup, Denmark) and phenotypically confirmed by immunochromatography using the NG-Test^®^ Carba 5 multiplex assay (NG Biotech, Guipry, France) to identify KPC, NDM, VIM, IMP, and OXA-48-like protein families. Subsequently, the specific carbapenemase-encoding genes and the clonal relationship of the isolates were confirmed by the Andalusian Laboratory of Molecular Typification within the PIRASOA program using whole-genome sequencing (WGS). Massive sequencing was performed on the Illumina platform (Illumina Inc., San Diego, CA, USA), and bioinformatic analysis was carried out using the CLC Genomics Workbench v10 (Qiagen, Iberia, Las Rozas de Madrid, Madrid, Spain). Determinants of resistance were characterized using the ResFinder (Lyngby, Denmark) and CARD (Hamilton, ON, Canada) databases, and strain characterization was performed as previously described [14].

The conventional antimicrobial agents evaluated in this study included gentamicin, tobramycin, amikacin, nitrofurantoin, fosfomycin, trimethoprim–sulfamethoxazole, and colistin. Antimicrobial susceptibility testing for conventional agents (gentamicin, tobramycin, amikacin, nitrofurantoin, and trimethoprim–sulfamethoxazole) was routinely carried out using the MicroScan^®^ WalkAway (Beckman Coulter, Brea, CA, USA) and VITEK^®^ 2 (bioMérieux, France) automated systems [12], with MIC values recorded for every isolate.

Aztreonam–avibactam susceptibility was determined by a gradient diffusion method using MIC Test Strips (bioMérieux, France). It was carried out using Mueller–Hinton agar plates (Becton Dickinson, Sparks, USA). The inoculum was standardized to a 0.5 McFarland turbidity using a densitometer (Beckman Coulter). After inoculation, the strips were applied and the plates were incubated at 37 °C for 24 h. MIC values were read at the point of 100% inhibition where the ellipse intersected the strip. Cefiderocol susceptibility was determined using the UMIC^®^ Cefiderocol broth microdilution system (Bruker, Bremen, Germany). Ready-to-use microdilution wells containing dehydrated cefiderocol concentrations ranging from 0.03 to 32 μg/mL were inoculated with iron-depleted cation-adjusted Mueller–Hinton broth provided by the manufacturer and incubated aerobically at 35 ± 2 °C for 24 h. The sample size was limited to 99 isolates for aztreonam–avibactam (54 Enterobacterales and 45 *Pseudomonas* spp.) and 111 isolates for cefiderocol (53 Enterobacterales and 58 *Pseudomonas* spp.), corresponding to commercial susceptibility testing systems available since 2021.

In our local hospital cohort, all antimicrobial susceptibility testing was strictly quantitative (MIC-based) using Etest^®^ for aztreonam–avibactam and broth microdilution (UMIC^®^) for cefiderocol; qualitative synergy assays were not used. To guarantee strict temporal comparability across the 10-year study period (2016–2025) and eliminate classification bias from evolving standards, raw MIC values for all conventional and novel antimicrobials were reinterpreted uniformly according to the latest EUCAST clinical breakpoint guidelines (v16.0, 2026), strictly adhering to species-specific applicability.

Specifically for conventional agents, nitrofurantoin clinical breakpoints (S ≤ 64 mg/L) were applied exclusively to *E. coli* and not extrapolated to non-*E. coli* Enterobacterales species, whereas fosfomycin breakpoints were restricted to Enterobacterales and omitted (reported as N/A) for *Pseudomonas* spp.

For novel agents, MIC values were interpreted according to 2026 EUCAST criteria: For cefiderocol, clinical breakpoints of Susceptible (S ≤ 2 mg/L) and Resistant (R > 2 mg/L) were applied for Enterobacterales and specifically for *P. aeruginosa* in accordance with species-specific EUCAST guidelines [13].

For aztreonam–avibactam, the updated EUCAST 2026 clinical breakpoint of S ≤ 4 mg/L and R > 4 mg/L(with avibactam at a fixed concentration of 4 mg/L) were used for Enterobacterales. In accordance with current EUCAST nomenclature and dosing guidelines, isolates categorized as ‘Susceptible, increased exposure’ (I) were grouped together with ‘Susceptible, standard dosing’ (S) isolates to calculate overall susceptibility rates, as clinical efficacy is maintained in both categories provided that systemic exposure is optimized. For *Pseudomonas* spp., as EUCAST 2026 explicitly notes insufficient evidence to establish clinical breakpoints for aztreonam–avibactam, MIC values were interpreted strictly against the epidemiological cut-off value (ECOFF = 16 mg/L) and reported as wild-type (WT ≤ 16 mg/L) versus non-wild-type (NWT > 16 mg/L) distributions rather than clinical susceptibility.

Colistin was tested via reference broth microdilution in accordance with EUCAST recommendations [13]. Manual microdilution was used in the UMic test (BioCentric, Bandol, France) in our laboratory in order to confirm the resistances that the MicroScan or Vitek2 systems has previously detected [12]. The possible MIC ranges were ≤0.06 to >64 mg/L for the manual microdilution.

Fosfomycin susceptibility was determined using the reference agar broth dilution method on Mueller–Hinton agar supplemented with 25 mg/L glucose-6-phosphate, or alternatively, via the automated VITEK^®^ 2 (bioMérieux) and MicroScan^®^ WalkAway (Beckman Coulter) panels. MIC values were interpreted according to EUCAST clinical breakpoints. For *E. coli*, the clinical breakpoint for oral treatment of uncomplicated urinary tract infections (susceptible ≤ 32 mg/L) was applied, whereas for non-*E. coli* Enterobacterales, MICs were evaluated considering the intravenous clinical breakpoint (susceptible ≤ 32 mg/L). For *Pseudomonas* spp., owing to the absence of official EUCAST clinical breakpoints and species-specific interpretative criteria, fosfomycin susceptibility criteria were not extrapolated, and isolates were reported as not applicable (N/A).

The total number of clinical isolates tested (denominators) varied across the evaluated antimicrobial agents due to several methodological and biological factors. First, the novel agents cefiderocol and aztreonam–avibactam were introduced into routine laboratory testing only from 2021 onwards, and were therefore evaluated exclusively on isolates from the second study period (2021–2025). Second, minor denominator variations occurred during retrospective testing due to the loss of cell viability in a small subset of stored historical isolates, which could not be successfully subcultured or revived from freezer storage. Third, certain conventional antimicrobials were not tested against specific bacterial groups due to intrinsic resistance or the absence of species-specific EUCAST clinical breakpoints (such as nitrofurantoin restricted exclusively to *E. coli*, and fosfomycin or trimethoprim–sulfamethoxazole omitted for *Pseudomonas* spp.) [13], as well as chronological changes in the commercial automated testing panels used by our laboratory over the 10-year study period.

Rigorous quality control (QC) protocols were performed throughout the study period to guarantee the accuracy and reproducibility of all susceptibility results. Standard reference strains obtained from the American Type Culture Collection (ATCC) and the National Collection of Type Cultures (NCTC) were utilized as control organisms: *E. coli* ATCC 25922, *P. aeruginosa* ATCC 27853, *K. pneumoniae* ATCC 700603, and the colistin-resistant mcr-1-positive *E. coli* NCTC 13846. Quality control testing was conducted weekly for routine automated platforms and on each day of testing (daily) whenever the novel agents (cefiderocol and aztreonam–avibactam) or reference broth microdilution assays were evaluated. Exact acceptable MIC ranges for control strains were monitored strictly according to EUCAST quality control guidelines (v14.0/v16.0):

Colistin: *E. coli* ATCC 25922 (acceptable range: 0.25–1 mg/L, target: 0.5 mg/L) and *E. coli* NCTC 13846 (mcr-1 positive; acceptable range: 2–8 mg/L, target: 4 mg/L);

Cefiderocol: *E. coli* ATCC 25922 (acceptable range: 0.06–0.5 mg/L) and *P. aeruginosa* ATCC 27853 (acceptable range: 0.12–1 mg/L), evaluated using iron-depleted cation-adjusted Mueller–Hinton broth (ID-CAMHB) supplied within the commercial UMIC^®^ system to simulate the required iron-limiting environment;

Aztreonam–avibactam: *E. coli* ATCC 25922 (acceptable range: 0.03–0.12 mg/L), *P. aeruginosa* ATCC 27853 (acceptable range: 1–8 mg/L), and *K. pneumoniae* ATCC 700603 (acceptable range: 0.03–0.25 mg/L).

In the event of any out-of-range QC value, clinical reporting was suspended, a full technical investigation of the batch was conducted, and the entire testing run was repeated. Clinical isolates’ MIC values were only validated and recorded once acceptable QC parameters were successfully restored.

### 2.4. Data Collection and Statistical Analysis

The following variables were collected from the laboratory information system (MODULAB^®^, Werfen, Spain), which is integrated into the electronic health record of the Andalusian Public Health System: patient age, sex, and the microorganism isolated from each urine culture. All data were anonymized prior to analysis. No clinical information regarding comorbidities, previous antimicrobial exposure, or other risk factors associated with specific microorganisms was available.

To ensure microbiological independence and avoid overrepresenting persistent or recurrent isolates, the analysis was strictly restricted to a single clinical isolate per patient (the first MBL-producing isolate recovered) across the entire 10-year study period. Consequently, the 182 included isolates correspond to exactly 182 unique patients.

Descriptive statistics are used to summarize antimicrobial susceptibility data. Proportions of susceptible isolates were calculated alongside exact 95% confidence intervals (95% CI) using the Clopper–Pearson exact binomial method. MIC ranges, MIC_50_ and MIC_90_ values were calculated and reported for each antimicrobial agent across both study periods.

Comparisons of susceptibility rates according to bacterial species and study period were performed using Pearson’s chi-square test or Fisher’s exact test, as appropriate according to sample size. To quantify effect sizes, unadjusted Odds Ratios (ORs) with 95% CIs were calculated for all pairwise comparisons. Pairwise comparisons were adjusted for multiple testing using the Benjamini–Hochberg false discovery rate (FDR) procedure.

All statistical analyses were performed using RStudio (version 2022.07.1) with the RVAideMemoire and rcompanion packages [15]. A two-sided *p*-value < 0.05 was considered statistically significant. Importantly, because this retrospective study was based exclusively on microbiological laboratory databases without access to patient-level clinical covariates and was limited by small sample sizes in certain subgroup strata, all comparative statistical analyses (including period-to-period and between-species comparisons) are exploratory and unadjusted, and their results should be interpreted cautiously as descriptive indicators of potential trends. To perform a direct head-to-head comparison and control for potential cohort bias, a sub-analysis was conducted on the matched-pair subset of isolates tested against both agents simultaneously using McNemar’s test for paired proportions.

For the systematic review, susceptibility and synergy data extracted from the 22 fully accessible studies (out of 23 eligible studies identified) were synthesized and reported exclusively as crude aggregated proportions (calculated as the total number of susceptible or synergistic isolates divided by the total number of isolates tested across all studies). A formal meta-analysis of proportions was intentionally not performed, due to the critical clinical, microbiological, and methodological heterogeneity observed among the included studies, such as variations in bacterial species, geographical locations, and susceptibility testing techniques (quantitative MICs versus qualitative synergy assays).

### 2.5. Ethical Considerations

The study was conducted in accordance with the principles of the Declaration of Helsinki (2024) and the ethical recommendations for epidemiological research [16].

This was a non-interventional study based exclusively on microbiological data generated during routine clinical practice. Biological specimens were processed solely for standard diagnostic purposes as requested by the attending physicians, and no additional sampling or modification of routine laboratory procedures was undertaken.

Data analysis was performed using a fully anonymized database. Individual patients were identified only by independent infectious episodes, with recurrent episodes considered distinct when they occurred at least six weeks apart.

Authorization to access and analyze the anonymized microbiological database was granted by the Clinical Microbiology Unit of Virgen de las Nieves University Hospital (Granada, Spain). According to Spanish legislation on personal data protection (Organic Law 3/2018 of 5 December on the Protection of Personal Data and Guarantee of Digital Rights), formal approval by an institutional ethics committee was not required, because no identifiable personal information was collected and the study involved no intervention or modification of routine clinical practice.

## 3. Results

### 3.1. Systematic Review

A total of 119 records were identified through PubMed. Following title/abstract screening, 96 records were excluded. Of the 23 reports sought for retrieval, 1 full-text article was unavailable; thus, 22 fully accessible studies met all inclusion criteria and were included in the qualitative synthesis (summarized in Table 1).

Overall, Enterobacterales constituted the predominant bacterial group, accounting for 70.0% (*n* = 1047) of all isolates included in the reviewed studies. Among these organisms, *K. pneumoniae* was the most frequently reported species, representing 12.3% of the total study population. The remaining 30.0% (*n* = 449) corresponded exclusively to *P. aeruginosa*, with no other *Pseudomonas* species reported in the selected literature.

Regarding the molecular epidemiology of metallo-β-lactamases within the systematic review database, New Delhi metallo-β-lactamase (NDM) was the most prevalent enzyme globally, accounting for 50.5% (*n* = 759) of these international isolates, followed by Verona integron-encoded metallo-β-lactamase (VIM), detected in 39.4% (*n* = 593). Imipenemase (IMP) was considerably less frequent, representing approximately 9.0% (*n* = 135) of isolates, while none of the included studies specifically reported the coproduction of additional carbapenemases. Conversely, as detailed in Section 3.3, VIM was the highly predominant resistance mechanism in our local clinical cohort, demonstrating a clear epidemiological divergence.

When disaggregating the testing methodologies, cefiderocol susceptibility was exclusively evaluated using quantitative MIC-based methods (mainly broth microdilution), yielding a crude aggregated susceptibility proportion of 58.1% across the quantitative studies. For aztreonam–avibactam, the results differed significantly depending on the assay type:

(i) In studies utilizing standardized quantitative MIC-based testing, aztreonam–avibactam demonstrated excellent activity against Enterobacterales, reaching a crude aggregated susceptibility proportion of 93.1%, with susceptibility rates in individual cohorts ranging between 90.0% and 100%. However, significant geographic and species-specific variability was noted, with quantitative susceptibility dropping to 51.3% in China [30] and as low as 27.0% in Germany [32] for *P. aeruginosa* isolates.
(ii) In studies utilizing qualitative phenotypic synergy assays (e.g., ceftazidime–avibactam plus aztreonam disks or crossing strips), the in vitro restoration of aztreonam activity was exceptionally high, with synergy/restoration rates ranging from 88.2% to 100% across the evaluated isolates [18,19,24,29,37]. These qualitative assays consistently demonstrated the biological plausibility of the combination, although they do not correspond to standardized clinical susceptibility breakpoints.

Notably, reduced cefiderocol activity against NDM-producing isolates was consistently reported across several recent studies [23,26]. Conversely, studies evaluating isolates co-producing multiple MBL [31,35] generally reported low MIC values for aztreonam–avibactam, supporting its preserved in vitro activity even in these highly resistant phenotypes.

Methodological quality assessment using the adapted JBI critical appraisal tool demonstrated an overall low-to-moderate risk of bias across the included studies. Sixteen studies (72.7%) exhibited a low risk of bias across all four evaluated domains. The remaining six studies (27.3%) were assigned a moderate risk of bias, primarily driven by Domain 3 due to reliance on qualitative synergy testing methods lacking established EUCAST or CLSI clinical breakpoints. Overall, all 22 articles met at least 75% of the applicable quality criteria, exhibiting well-defined isolate selection, adherence to standardized susceptibility testing protocols, and molecular confirmation of underlying carbapenemase genotypes. Detailed study-level domain evaluations are provided in Supplementary Material S1 (Table S1).

### 3.2. Local Antimicrobial Susceptibility Study

A total of 182 clinical isolates producing metallo-β-lactamases (MBLs) were included in the local susceptibility analysis.

The annual distribution of isolates according to sex, age, and requesting healthcare setting (Emergency Department/Outpatient vs. Inpatient Ward) is presented in Table 2. A progressive increase in the number of isolates was observed between 2023 and 2025, with the highest annual count of detected isolates recorded in 2025.

Overall, most isolates were recovered from inpatient care settings (134/182, 73.6%). The highest proportion of cases occurred in patients aged 15–65 years (93/182, 51.1%), and males represented the majority of the study population (119/182, 65.4%).

The distribution of isolates according to hospital department is summarized in Table 3. The highest number of cases was identified in the Internal Medicine Department, accounting for 76 isolates (41.2%).

The annual distribution of MBL-producing Gram-negative bacteria is shown in Table 4. Consistent with the findings of the systematic review, Enterobacterales constituted the predominant bacterial group (94/182, 51.6%), with *K. pneumoniae* representing the most frequently isolated species (43/94, 45.7%). Among the *Pseudomonas* genus, *P. aeruginosa* was the highly predominant species, accounting for 91.9% of the group (79/86 isolates; 43.4% of the total cohort), while *Pseudomonas putida* represented the remaining 8.1% (7/86 isolates) (Table 4). This confirms that *P. aeruginosa* was the primary driver of *Pseudomonas* spp.-related infections in our clinical setting, demonstrating a microbiological distribution comparable to that reported in the international literature.

### 3.3. Distribution of Carbapenemase Genes

The annual distribution of carbapenemase genes is summarized in Table 5.

Across the total cohort of 182 MBL-producing clinical isolates, a single carbapenemase gene was identified in 138 isolates (75.8%), whereas 44 isolates (24.2%) harbored multiple carbapenemase determinants, including 5 dual-MBL producers (NDM + IMP) and 39 isolates co-producing an MBL alongside a class A or D serine carbapenemase (Table 5).

Accounting for both single-gene carriers and combination profiles across all 182 isolates, bla_VIM_ was the most prevalent MBL gene overall, present in 117 isolates (107 single producers, 8 VIM + OXA-48, 1 VIM + KPC, and 1 VIM + KPC + OXA-48). bla_NDM_ was identified in 48 isolates (14 single producers, 5 NDM + IMP, and 29 NDM + OXA-48), while bla_IMP_ was harbored by 22 isolates (17 single producers and 5 NDM + IMP). Among co-expressed serine carbapenemases, bla_OXA-48_-like determinants were detected in 38 isolates and bla_KPC_ in 2 isolates.

### 3.4. Antimicrobial Susceptibility

Antimicrobial susceptibility results, including susceptibility rates and MIC50 values for Enterobacterales and *Pseudomonas* spp., are summarized in Table 6 for both study periods (2016–2020 and 2021–2025). Exploratory, unadjusted statistical comparisons according to bacterial group and study period are detailed in Table 7.

Because cefiderocol and aztreonam–avibactam were incorporated into routine clinical testing protocols only from 2021 onwards, susceptibility evaluations for these two novel agents were performed exclusively during the second study period (2021–2025).

Among the novel antimicrobial agents, aztreonam–avibactam demonstrated exceptional in vitro activity, achieving complete susceptibility (100%, 54/54; MIC_50_ = 0.125 mg/L) against MBL-producing Enterobacterales under updated EUCAST 2026 clinical breakpoints (S ≤ 4 mg/L). Against *Pseudomonas* spp., for which EUCAST 2026 explicitly notes insufficient evidence for clinical breakpoints, aztreonam–avibactam exhibited a 77.8% WT phenotype rate (35/45; MIC_50_ = 8 mg/L) based on the ECOFF. In contrast, cefiderocol exhibited excellent in vitro activity against MBL-producing *Pseudomonas* spp., with a susceptibility rate of 86.2% (50/58; MIC_50_ = 1 mg/L). However, its activity was considerably lower against Enterobacterales, with only 39.6% (21/53) of the isolates classified as susceptible (MIC_50_ = 4 mg/L). In the exploratory head-to-head comparisons between these species, both differences were highly significant after adjusting for multiple testing: aztreonam–avibactam was significantly more active against Enterobacterales (raw *p* = 0.0002, FDR-adjusted *p* = 0.0010; OR = 32.24, 95% CI: 1.83–567.70), whereas cefiderocol was significantly more active against *Pseudomonas* spp. (raw *p* < 0.0001, FDR-adjusted *p* < 0.0001; OR = 0.10, 95% CI: 0.04–0.27). Regarding conventional antimicrobial agents, gentamicin exhibited highly limited in vitro activity. No susceptible *Pseudomonas* spp. isolates were identified during the first study period (0/9, 0%), and only 12.9% (9/70) were susceptible during the second period, representing a non-significant temporal change (raw *p* = 0.2014, FDR-adjusted *p* = 0.6850; OR = 0.34, 95% CI: 0.02–6.35). Among Enterobacterales, gentamicin susceptibility decreased significantly over time, dropping from 62.5% (20/32) in the 2016–2020 period to 32.8% (20/61) in the 2021–2025 period (raw *p* = 0.0068, FDR-adjusted *p* = 0.0286; OR = 3.42, 95% CI: 1.40–8.35). Similarly, tobramycin demonstrated extremely poor activity throughout the ten-year study, with susceptibility rates remaining below 20% irrespective of the bacterial group or study period (Enterobacterales: 15.6% vs. 15.0%, raw *p* = 0.9421, FDR-adjusted *p* = 1.0000, OR = 1.05, 95% CI: 0.29–3.81; *Pseudomonas* spp.: 11.1% vs. 18.8%, raw *p* = 0.5841, FDR-adjusted *p* = 1.0000, OR = 0.54, 95% CI: 0.06–4.69).

Among the aminoglycosides evaluated, amikacin showed a more favorable in vitro susceptibility profile. In Enterobacterales, susceptibility increased significantly from 60.0% (12/20) during the first study period to 88.1% (52/59) during the second period (raw *p* = 0.0076, FDR-adjusted *p* = 0.0475; OR = 0.20, 95% CI: 0.06–0.67). A similar upward trend was descriptively observed among *Pseudomonas* spp., where amikacin susceptibility increased from 33.3% (3/9) to 47.2% (25/53), although this change did not reach statistical significance after correcting for multiple comparisons (raw *p* = 0.4510, FDR-adjusted *p* = 0.6293; OR = 0.56, 95% CI: 0.13–2.48).

Due to intrinsic resistance mechanisms and the absence of established EUCAST clinical breakpoints, nitrofurantoin was not evaluated against *Pseudomonas* spp. Among Enterobacterales as a whole, evaluated using the *E. coli* clinical breakpoint (S ≤ 64 mg/L) as an exploratory surrogate threshold from automated laboratory panels, nitrofurantoin susceptibility experienced a significant decline over time, falling from 65.6% (21/32; MIC_50_ = 64 mg/L) during the first study period to 20.0% (8/40; MIC_50_ > 64 mg/L) during the second period (raw *p* = 0.0001, FDR-adjusted *p* = 0.0008; OR = 7.64, 95% CI: 2.63–22.13). Note that official EUCAST clinical interpretation remains restricted exclusively to *E. coli*.

Fosfomycin also exhibited reduced in vitro potency among Enterobacterales over the study periods. During the 2016–2020 period, susceptibility was 75.0% (24/32), decreasing to 52.6% (20/38) during the 2021–2025 period. Although this temporal reduction was borderline in unadjusted analyses, it did not retain statistical significance after FDR adjustment (raw *p* = 0.0512, FDR-adjusted *p* = 0.1908; OR = 2.70, 95% CI: 0.97–7.51). In accordance with EUCAST guidelines, fosfomycin susceptibility was not evaluated for *Pseudomonas* spp. due to the absence of established clinical breakpoints. As expected, trimethoprim–sulfamethoxazole (TMP-SMX) exhibited no in vitro activity against *Pseudomonas* spp. due to intrinsic resistance mechanisms (0/9 in period 1 and 0/40 in period 2). Among Enterobacterales, susceptibility remained critically low throughout the entire study, reaching only 3.1% (1/32) in the first period and a non-significant increase to 14.5% (9/62) in the second period (raw *p* = 0.1512, FDR-adjusted *p* = 0.2519; OR = 0.19, 95% CI: 0.02–1.57). Finally, among all conventional antimicrobials, colistin demonstrated the highest and most sustained overall in vitro activity. Susceptibility among Enterobacterales was 100% (20/20) and 87.8% (36/41) during the first and second study periods, respectively (raw *p* = 0.0782, FDR-adjusted *p* = 0.2519; OR = 6.18, 95% CI: 0.32–117.48). Corresponding susceptibility rates for *Pseudomonas* spp. remained high and stable at 77.8% (7/9) during the first period and 88.2% (60/68) during the second period, showing no statistically significant temporal changes (raw *p* = 0.3951, FDR-adjusted *p* = 0.4629; OR = 0.47, 95% CI: 0.08–2.65).

### 3.5. Stratified Susceptibility Analysis

To gain deeper microbiological insights, susceptibility profiles for the novel agents were stratified by key sub-variables, including species, MBL enzyme type, and carbapenemase co-production status.

#### 3.5.1. Stratification by Key Species

The distribution of specific MBL enzymes and co-produced serine-carbapenemases across bacterial species is detailed in Table 8. Overall, *K. pneumoniae* was the predominant species among Enterobacterales (45.7%, 43/94), followed by the *Enterobacter cloacae complex* (36.2%, 34/94), while *P. aeruginosa* represented the vast majority of *Pseudomonas* spp. isolates (91.9%, 79/86).

In this species-stratified analysis, aztreonam–avibactam demonstrated complete in vitro activity (100% susceptibility, 54/54) against MBL-producing Enterobacterales, with 100% of tested *K. pneumoniae* and *E. coli* isolates being fully susceptible (Table 8). For *P. aeruginosa*, aztreonam–avibactam exhibited a 77.8% wild-type (WT) phenotype rate (35/45) based on the EUCAST ECOFF threshold. Conversely, cefiderocol displayed highly contrasting species-specific results: it was highly potent against *P. aeruginosa* (86.2% susceptibility, 50/58) but showed compromised in vitro activity against Enterobacterales, which were largely dominated by *K. pneumoniae* (39.6% susceptibility, 21/53).

#### 3.5.2. Stratification by MBL Type (VIM vs. NDM)

The activity profiles of the novel agents were strongly influenced by the specific MBL family (Table 9). In our institution, the VIM family was predominant (*n* = 107) and was primarily associated with *Pseudomonas* spp. (*n* = 86), alongside 21 Enterobacterales. Cefiderocol demonstrated high in vitro susceptibility against these VIM-producing isolates (86.2%, 50/58 in *Pseudomonas* spp. and 92.3%, 12/13 in Enterobacterales), compared to a 77.8% WT rate (35/45) for aztreonam–avibactam in *Pseudomonas* spp. based on its EUCAST ECOFF. Conversely, single NDM-producing isolates (*n* = 14) and IMP-producing isolates (*n* = 17) were identified among Enterobacterales and *Aeromonas* spp. For NDM-producing strains, aztreonam–avibactam achieved complete in vitro susceptibility (100%, 7/7), whereas cefiderocol susceptibility was markedly reduced (28.6%, 2/7) (Table 9), a finding that aligns with the international literature compiled in our systematic review.

#### 3.5.3. Stratification by Co-Production Status (Single MBL vs. Multiple Carbapenemases)

In the local cohort, 138 isolates produced a single MBL (predominantly VIM in *Pseudomonas* spp., and VIM, NDM, or IMP in Enterobacterales), while 44 isolates exhibited complex carbapenemase profiles, including 5 dual-MBL producers (NDM + IMP) and 39 co-producers of an MBL alongside a serine-carbapenemase (predominantly NDM + OXA-48, *n* = 29, and VIM + OXA-48, *n* = 8) (Table 8 and Table 9).

Co-production status had a major impact on drug performance (Table 9). Aztreonam–avibactam maintained complete in vitro activity (100.0% susceptibility, 26/26) against these complex co-producing Enterobacterales (both dual MBLs and MBL + serine-carbapenemase producers). Mechanistically, this high potency is explained by the ability of avibactam to inhibit co-existing OXA-48-like or KPC serine-carbapenemases, thereby restoring the activity of aztreonam, which is intrinsically stable against MBL hydrolysis. In sharp contrast, cefiderocol susceptibility dropped dramatically to 11.5% (3/26) among all co-producing Enterobacterales, reaching its lowest point in NDM + OXA-48 co-producers (9.5%, 2/21) (Table 9).

#### 3.5.4. Head-to-Head Matched-Pair Analysis

Because aztreonam–avibactam and cefiderocol in vitro susceptibility testing was performed on overlapping cohorts of isolates from the 2021–2025 period (owing to retrospective recovery and viability variations), a direct matched-pair analysis was conducted on the subset of isolates successfully evaluated against both agents.

Among Enterobacterales, 53 isolates were successfully tested against both drugs; aztreonam–avibactam demonstrated 100% in vitro susceptibility (53/53) compared to 39.6% (21/53) for cefiderocol, a difference that was highly significant (McNemar’s *p* < 0.001). Among *Pseudomonas* spp., 45 isolates were evaluated against both agents; in vitro susceptibility to cefiderocol was 86.7% (39/45) compared to a 77.8% wild-type phenotype rate (35/45) for aztreonam–avibactam based on its EUCAST ECOFF, showing no statistically significant difference in this matched cohort (McNemar’s *p* = 0.344).

## 4. Discussion

The present study evaluated the in vitro activity of two promising novel antimicrobial agents currently available against MBL-producing Gram-negative bacteria, namely aztreonam–avibactam and cefiderocol. By combining a systematic review of the international literature with a ten-year epidemiological analysis from a tertiary-care hospital, this study provides an integrated overview of the current susceptibility patterns of these last-line agents while highlighting the influence of local molecular epidemiology on their performance.

In parallel, the susceptibility profiles of conventional antimicrobial agents were evaluated to establish baseline activity patterns and identify older agents retaining in vitro susceptibility against MBL-producing isolates. One of the most relevant findings was the marked increase in the number of MBL-producing isolates recovered during the study period, rising from only three isolates in 2016 to 81 isolates in 2025. This trend mirrors the global expansion of carbapenem-resistant Gram-negative bacteria and reinforces the growing clinical importance of these pathogens in routine hospital practice.

The systematic review revealed a clear predominance of NDM-producing isolates, whereas VIM was the most prevalent metallo-β-lactamase identified in our institution (Table 8). This difference in molecular epidemiology has important therapeutic implications because susceptibility to novel β-lactam agents is strongly influenced by the underlying resistance mechanism (Table 9). In particular, while cefiderocol demonstrated high in vitro activity against VIM producers, its susceptibility dropped sharply against single NDM producers (28.6%, 2/7) and was severely compromised among complex co-producers—reaching its lowest point in NDM + OXA-48 Enterobacterales (9.5%, 2/21; overall co-producer susceptibility: 11.5%, 3/26). Conversely, aztreonam–avibactam retained complete in vitro activity (100.0%, 26/26) against these complex co-producing phenotypes. This mechanism-dependent limitation explains the discrepancies observed between our local results and the crude aggregated international data [39]. Indeed, although the overall susceptibility to cefiderocol identified in the systematic review was moderate, our local experience demonstrated high in vitro susceptibility specifically against *P. aeruginosa*, whereas activity against Enterobacterales remained considerably lower (39.6% overall, dropping to 11.5% in co-producers). These findings suggest that the clinical usefulness of cefiderocol should be interpreted within the context of the local epidemiology of carbapenemase gene distribution and species-specific activity rather than relying exclusively on international susceptibility data. Conversely, aztreonam–avibactam demonstrated the most consistent in vitro activity throughout our study. Complete in vitro susceptibility was observed among Enterobacterales, including complex co-producers, whereas against Pseudomonas spp., for which EUCAST 2026 explicitly notes insufficient evidence to establish clinical breakpoints, aztreonam–avibactam exhibited a 77.8% WT phenotype rate based on its ECOFF. These results are consistent with most published studies included in the systematic review, which generally reported high in vitro susceptibility rates above 90%, although important variability exists across different geographical regions. The markedly lower in vitro susceptibility reported by Wu et al. illustrates how regional epidemiology and bacterial population structure may substantially influence antimicrobial performance.

Taken together, these observations reinforce the importance of continuous local microbiological surveillance. Because the prevalence of carbapenemase genes differs considerably among institutions and countries, treatment recommendations based solely on international evidence may not accurately reflect local susceptibility patterns.

Beyond the evaluation of novel agents, our findings also highlight the retained in vitro activity of selected conventional antimicrobials. Colistin demonstrated the highest overall susceptibility among traditional agents, maintaining activity against both Enterobacterales and *Pseudomonas* spp. throughout the study period. Likewise, amikacin showed a significant improvement in susceptibility among Enterobacterales during the second study period. While in vitro susceptibility to selected conventional agents was retained, whether this translates into real-world clinical utility remains to be validated through clinical outcome analyses. By contrast, conventional agents were interpreted strictly adhering to species-specific EUCAST limitations: nitrofurantoin clinical breakpoints apply exclusively to *E. coli* and were not extrapolated to non-*E. coli* Enterobacterales, whereas nitrofurantoin, fosfomycin, and trimethoprim–sulfamethoxazole were classified as Not Applicable for *Pseudomonas* spp. due to intrinsic resistance or the absence of established clinical breakpoints. Consequently, these agents are not active in vitro against MBL-producing *Pseudomonas* isolates or selected non-*E. coli* Enterobacterales; thus, restricting susceptibility reporting to target species helps align laboratory reporting with their established spectrum of activity.

Overall, our findings demonstrate that the choice between cefiderocol and aztreonam–avibactam should be guided not only by antimicrobial susceptibility testing and species-specific interpretative criteria, but also by the local distribution of carbapenemase genes. This reinforces the importance of continuous local microbiological surveillance to monitor in vitro activity and susceptibility trends. However, translating these laboratory findings into clinical practice guidelines requires robust clinical trials and real-world validation, as in vitro potency does not always reflect clinical efficacy or prevent resistance selection in vivo.

## 5. Study Limitations

This study has several limitations that should be acknowledged. Regarding the systematic review, while an expanded search strategy was conducted using Pubmed/Medline, the possibility of missing relevant studies cannot be completely eliminated. Furthermore, although 23 studies met our initial eligibility criteria, one study was not accessible in full-text despite extensive library searches and institutional retrieval efforts, thereby limiting our final data extraction and quality appraisal to 22 fully accessible articles.

First, regarding our local susceptibility study, because cefiderocol and aztreonam–avibactam were incorporated into routine laboratory testing only from 2021 onwards, their in vitro activity could not be evaluated throughout the entire ten-year study period, limiting the assessment of long-term susceptibility trends. Furthermore, the variation in the number of isolates tested across different antimicrobial agents introduces a potential selection bias. This variation was primarily driven by the retrospective viability of stored isolates; older isolates from the first study period (2016–2020) were more prone to viability loss during cryogenic recovery, which occasionally prevented their successful subculture and subsequent susceptibility testing against the novel agents or certain conventional panels. While this could theoretically skew findings towards more robust or recently recovered strains, the impact on our overall susceptibility rates is expected to be minimal, as the vast majority of clinically significant MBL-producing isolates were successfully retrieved and analyzed. Additionally, because of these retrieval limitations, cefiderocol and aztreonam–avibactam were tested on overlapping rather than completely identical paired cohorts, making overall comparisons between the two agents partially indirect. Although a matched-pair sub-analysis was performed on the subset of isolates evaluated against both drugs to mitigate this, caution should be exercised when comparing their overall descriptive rates.

Second, the evaluation of certain conventional antimicrobials was constrained by species-specific EUCAST limitations. Specifically, nitrofurantoin susceptibility could not be extrapolated to non-*E. coli* Enterobacterales, and fosfomycin susceptibility was omitted for *Pseudomonas* spp. due to the absence of established clinical breakpoints, limiting comparative long-term trend analysis for these specific agent-organism combinations. Third, the systematic literature search was restricted exclusively to PubMed/MEDLINE. Although PubMed represents the primary, universally recognized benchmark repository in biomedical research, relying on a single database may theoretically omit relevant publications indexed in secondary databases. Furthermore, potential publication and reporting biases inherent to in vitro susceptibility literature cannot be entirely ruled out.

Fourth, this was a single-center retrospective study based exclusively on urinary isolates. Consequently, caution should be exercised when extrapolating these findings to other healthcare settings or to infections involving different anatomical sites.

Finally, due to the retrospective, laboratory-based design of our local study, we lacked access to patient-level clinical variables (such as comorbidities, intensive care unit stay, or prior antimicrobial treatment duration). Consequently, we could not perform multivariable logistic regression or generalized estimating equations (GEEs) to adjust our comparative analyses for potential clinical confounding factors, and our comparative statistics remain exploratory and unadjusted. Future prospective, multicenter studies incorporating clinical registries are required to perform adjusted analyses and validate these preliminary microbiological findings.

## 6. Conclusions

This combined systematic review and local epidemiological study demonstrates that both cefiderocol and aztreonam–avibactam exhibit substantial in vitro activity against MBL-producing Gram-negative bacteria, although important differences were observed according to bacterial species and underlying carbapenemase genes.

Aztreonam–avibactam showed the highest overall in vitro activity, with complete susceptibility among Enterobacterales including complex co-producers—and a high wild-type phenotype rate against *Pseudomonas* spp. based on its EUCAST ECOFF threshold. In contrast, cefiderocol demonstrated excellent in vitro activity against *Pseudomonas* spp. but considerably lower susceptibility among Enterobacterales (39.6% overall, dropping to 11.5% in co-producers), confirming that its in vitro spectrum of activity depends heavily on the specific MBL family and the local distribution of co-existing serine-carbapenemases.

Among conventional antimicrobials, colistin maintained the highest in vitro susceptibility rates throughout the study period, while amikacin retained notable activity against selected MBL-producing Enterobacterales. Overall, these findings highlight the critical role of continuous local surveillance of carbapenemase epidemiology and in vitro susceptibility to inform local empirical treatment guidelines. However, because our study is strictly based on laboratory susceptibility testing, these in vitro observations cannot be directly extrapolated to clinical success. Prospective clinical trials and real-world comparative studies are required to validate these findings and to evaluate the true clinical efficacy and patient outcomes of both novel and conventional antimicrobial regimens against MBL-producing pathogens.

## Figures and Tables

**Table 1:** Studies included (*n* = 22) in the systematic review of cefiderocol and aztreonam–avibactam.

Reference	Study Population (*n*)	Carbapenemase Mechanism	Main Findings	Main Conclusion
[17]	471 Enterobacterales and *Pseudomonas* spp.	75 NDM, 62 IMP, 334 VIM	AZT–AVI: MICs ≤ 0.015–8 μg/mL in Enterobacterales	Variable results; difficult clinical interpretation
[18]	1 Enterobacterales 2 *Pseudomonas* spp.	1 NDM, 1 IMP, 1 VIM	AZT–AVI: 66.7% synergy	Synergy demonstrated in 2 of 3 isolates
[19]	52 Enterobacterales	47 NDM, 5 VIM	AZT–AVI: 100% synergy	Excellent activity in bloodstream isolates
[20]	17 Enterobacterales	NDM/IMP	AZT–AVI: high synergy (MIC ≤ 0.125/4–2/4 μg/mL)	High inhibitory activity
[21]	4 Enterobacterales	IMP	AZT–AVI: 100% synergy	Complete in vitro activity in clinical cases
[22]	1 Enterobacterales	VIM	AZT–AVI: 100% synergy	Synergy confirmed
[23]	49 Enterobacterales and *Pseudomonas* spp.	40 VIM, 9 NDM	AZT–AVI: susceptibility confirmed by synergy; CEF susceptibility: VIM-Enterobacterales 93.8%, VIM-*Pseudomonas* 87.5%, NDM 11.1%	Cefiderocol showed poor activity against NDM-producing isolates
[24]	15 Enterobacterales 2 *Pseudomonas* spp.	VIM	AZT–AVI: 88.2% synergy	Synergy observed in 15/17 isolates
[25]	143 Enterobacterales	74 NDM, 42 IMP, 27 VIM	AZT–AVI: 95.8% susceptibility; CEF: 86% susceptibility	Both antimicrobials showed high activity
[26]	343 Enterobacterales	328 NDM, 15 VIM	AZT–AVI: 99.7% synergy; CEF: 33.2% susceptibility	Cefiderocol showed limited activity against NDM
[27]	13 Enterobacterales and *Pseudomonas* spp.	VIM	AZT–AVI: synergy in 4 tested isolates; CEF: 92.3% susceptibility	Cefiderocol demonstrated high activity against VIM
[28]	15 *Pseudomonas* spp.	VIM	CEF: 93.3% susceptibility	Cefiderocol remained highly active
[29]	59 Enterobacterales	14 NDM, 12 IMP	AZT–AVI: 100% synergy	Consistent synergistic activity
[30]	121 Enterobacterales and *Pseudomonas* spp.	98 NDM, 11 VIM, 12 IMP	AZT–AVI susceptibility: Enterobacterales 90%; *Pseudomonas* spp. 51.3%	Reduced activity against *Pseudomonas* spp. in China
[31]	2 Enterobacterales	VIM + NDM	AZT–AVI and CEF: high susceptibility	Active despite coproduction of carbapenemases
[32]	63 *Pseudomonas* spp.	61 VIM, 2 NDM	AZT–AVI: 27% susceptibility	Significant resistance among German VIM isolates
[33]	5 *Pseudomonas* spp.	3 VIM, 2 IMP	AZT–AVI MIC: 4 to >64 mg/L; CEF MIC: 0.5–4 mg/L	Cefiderocol showed greater inhibitory activity
[34]	8 *Pseudomonas* spp.	8 VIM	AZT–AVI MIC: 4–16 mg/L; CEF MIC: 0.12–0.5 mg/L	Cefiderocol showed greater inhibitory activity
[35]	3 Enterobacterales	VIM + NDM	AZT–AVI MIC: 1–2 mg/L; CEF susceptible in 2/3 isolates	Therapeutic complexity due to carbapenemase coproduction
[36]	22 *Pseudomonas* spp.	21 VIM, 1 NDM	AZT–AVI MIC ≤ 16 mg/L	MICs close to the epidemiological cutoff
[37]	77 Enterobacterales	76 NDM, 1 NDM + VIM	AZT–AVI: 100% synergy; CEF: 75% susceptibility	Aztreonam–avibactam outperformed cefiderocol
[38]	43 Enterobacterales	28 NDM, 15 VIM	AZT–AVI susceptibility: NDM 91%, VIM 100%; CEF susceptibility: NDM 85%, VIM 87%	Excellent activity for both antimicrobials

Note: Susceptibility and synergy rates reported in Table 1 reflect the original interpretive criteria and methodologies defined by the study authors. Under current EUCAST 2026 guidelines (v16.0), clinical breakpoints for aztreonam–avibactam are S ≤ 4 mg/L for Enterobacterales, whereas values for *Pseudomonas* spp. are interpreted against the Epidemiological Cut-off Value (ECOFF = 16 mg/L, wild-type) due to insufficient evidence for clinical breakpoints.

**Table 2:** Characteristics of patients with carbapenemase-producing Gram-negative bacteria isolated, according to study period, sex, age, and requesting healthcare setting.

Study Period	Sex/Origin	≤14 Years	15–65 Years	>65 Years	Total
2016–2020	Female	–	8	7	15
	Male	–	18	8	26
	Emergency Department and Outpatient setting	–	9	5	14
	Inpatient Ward setting	–	17	10	27
2021–2025	Female	–	12	36	48
	Male	1	55	37	93
	Emergency Department and Outpatient setting	–	18	16	34
	Inpatient Ward setting	1	49	57	107

**Table 3:** Annual distribution of isolates according to hospital department.

Hospital Department	2016	2017	2018	2019	2020	2021	2022	2023	2024	2025
Internal Medicine (*n* = 76)	2	4	4	6	7	2	3	4	17	27
Hematology and Oncology (*n* = 31)	1	–	–	1	2	–	2	7	6	12
Intensive Care Unit (*n* = 20)	–	1	1	–	4	2	–	–	1	11
Emergency Department (*n* = 33)	–	1	–	2	1	1	1	2	8	17
Surgery (*n* = 22)	–	–	1	1	1	–	–	3	2	14

**Table 4:** Annual distribution and absolute counts of MBL-producing Gram-negative bacteria.

Microorganism	Species	2016	2017	2018	2019	2020	2021	2022	2023	2024	2025
Enterobacterales (*n* = 94)	*K. pneumoniae* (*n* = 43)	1	1	–	5	4	1	1	1	13	16
	*K. oxytoca* (*n* = 3)	1	–	1	–	–	–	–	1	–	–
	*E. cloacae* (*n* = 24)	1	5	1	3	7	–	–	2	3	2
	*E. bugandensis* (*n* = 2)	–	–	–	–	–	–	–	2	–	–
	*E. hormaechei* (*n* = 6)	–	–	–	–	–	–	–	1	–	5
	*E. asburiae* (*n* = 2)	–	–	–	–	–	–	–	–	2	–
	*E. coli* (*n* = 6)	–	1	–	–	–	–	–	–	3	2
	*C. freundii* (*n* = 7)	–	–	–	–	1	1	–	–	5	–
	*R. ornithinolytica* (*n* = 1)	–	–	–	–	–	–	–	–	–	1
*Pseudomonas* spp. (*n* = 86)	*P. aeruginosa* (*n* = 79)	–	–	3	2	3	3	3	8	7	50
	*P. putida* (*n* = 7)	–	–	1	–	–	–	1	1	–	4
*Aeromonas* spp. (*n* = 2)	*A. hydrophila* (*n* = 1)	–	–	–	–	–	–	–	–	1	–
	*A. caviae* (*n* = 1)	–	–	–	–	–	–	–	–	–	1

**Table 5:** Distribution of resistance mechanisms by study period.

Carbapenemase(s)	2016–2020	2021–2025
VIM (N = 107)	25	82
IMP (N = 17)	5	12
NDM (N = 14)	7	7
NDM + IMP (N = 5)	2	3
NDM + OXA-48 (N = 29)	1	28
VIM + OXA-48 (N = 8)	1	7
VIM + KPC (N = 1)	–	1
VIM + KPC + OXA-48 (N = 1)	–	1

**Table 6:** *In vitro* antimicrobial susceptibility proportions, exact 95% confidence intervals, and MIC distribution parameters (MIC50 and MIC90) for Enterobacterales and *Pseudomonas* spp. across study periods.

Antimicrobial Agent	Bacterial Group and Period	Sample Size (N)	Susceptibility n/N (%)	Exact 95% CI (%) ^a^	MIC_50_ (mg/L)	MIC_90_ (mg/L)
Cefiderocol	Enterobacterales (2021–2025)	53	21/53 (39.6%)	26.5–54.0%	4	8
	*Pseudomonas* spp. (2021–2025)	58	50/58 (86.2%)	74.6–93.9%	1	8
Aztreonam-avibactam	Enterobacterales (2021–2025)	54	54/54 (100%)	93.4–100.0%	0.125	0.5
	*Pseudomonas* spp. (2021–2025)	45	35/45 (77.8%)	62.9–88.8%	8	24
Gentamicin	Enterobacterales (2016–2020)	32	20/32 (62.5%)	43.7–78.9%	≤2	>8
	Enterobacterales (2021–2025)	61	20/61 (32.8%)	21.3–46.0%	>4	≥16
	*Pseudomonas* spp. (2016–2020)	9	0/9 (0.0%)	0.0–33.6%	>8	>8
	*Pseudomonas* spp. (2021–2025)	70	9/70 (12.9%)	6.1–23.0%	>8	≥16
Tobramycin	Enterobacterales (2016–2020)	32	5/32 (15.6%)	5.3–32.8%	>4	>8
	Enterobacterales (2021–2025)	40	6/40 (15.0%)	5.7–29.8%	>4	>4
	*Pseudomonas* spp. (2016–2020)	9	1/9 (11.1%)	0.3–48.2%	>8	>8
	*Pseudomonas* spp. (2021–2025)	69	13/69 (18.8%)	10.4–30.1%	>8	≥16
Amikacin	Enterobacterales (2016–2020)	20	12/20 (60.0%)	36.1–80.9%	≤8	>16
	Enterobacterales (2021–2025)	59	52/59 (88.1%)	77.1–95.1%	≤8	16
	*Pseudomonas* spp. (2016–2020)	9	3/9 (33.3%)	7.5–70.1%	16	>32
	*Pseudomonas* spp. (2021–2025)	53	25/53 (47.2%)	33.3–61.4%	16	>32
Nitrofurantoin *	Enterobacterales (2016–2020)	32	21/32 (65.6%)	46.8–81.4%	64	>64
	Enterobacterales (2021–2025)	40	8/40 (20.0%)	9.1–35.6%	≤64	>64
Fosfomycin	Enterobacterales (2016–2020)	32	24/32 (75.0%)	56.6–88.5%	≤32	>64
	Enterobacterales (2021–2025)	38	20/38 (52.6%)	35.8–69.0%	32	128
Trimethoprim–sulfamethoxazole	Enterobacterales (2016–2020)	32	1/32 (3.1%)	0.1–16.2%	>4/76	>4/76
	Enterobacterales (2021–2025)	62	9/62 (14.5%)	6.9–25.8%	>4/76	≥320
Colistin	Enterobacterales (2016–2020)	20	20/20 (100.0%)	83.2–100.0%	≤2	≤2
	Enterobacterales (2021–2025)	41	36/41 (87.8%)	73.8–95.9%	≤2	4
	*Pseudomonas* spp. (2016–2020)	9	7/9 (77.8%)	40.0–97.2%	≤2	4
	*Pseudomonas* spp. (2021–2025)	68	60/68 (88.2%)	78.1–94.8%	≤2	≥8

CI: Confidence Interval; MIC: Minimum Inhibitory Concentration; MIC50 and MIC90: MIC values inhibiting 50% and 90% of isolates, respectively. ^a^ Exact 95% binomial confidence intervals calculated using the Clopper–Pearson exact method. According to EUCAST 2026 guidelines, clinical breakpoints are not established for aztreonam–avibactam against *Pseudomonas* spp., due to insufficient evidence; values were categorized based on the Epidemiological Cut-off Value (ECOFF = 16 mg/L) as Wild-Type (WT ≤ 16 mg/L) vs. Non-Wild-Type (NWT > 16 mg/L). * EUCAST clinical breakpoints for nitrofurantoin (S ≤ 64 mg/L) apply strictly to *E. coli* and are not established for other Enterobacterales species or *Pseudomonas* spp. Consequently, official clinical susceptibility categories cannot be assigned to non-*E. coli* isolates. Sample sizes (*N*) vary across agents due to the introduction of novel drugs from 2021 onwards, loss of viability in stored historical isolates during retrospective recovery, species-specific EUCAST restrictions, and changes in commercial automated panel configurations over time.

**Table 7:** Statistical analysis of antimicrobial susceptibility according to microorganism and study period.

Antimicrobial	Comparison	Sample Size (*N*)	Results	Raw *p*-Value	FDR-Adjusted *p*-Value (*q*-Value)	OR (95% CI)	Interpretation
Cefiderocol	Enterobacterales vs. *Pseudomonas* spp.	111	21/53 vs. 50/58	<0.0001	**<0.0001**	0.10 (0.04–0.27)	Much higher activity against *Pseudomonas* spp.
Aztreonam–avibactam	Enterobacterales vs. *Pseudomonas* spp.	99	54/54 vs. 35/45	0.0002	**0.0010**	32.24 (1.83–567.70)	Higher activity against Enterobacterales
Gentamicin	Enterobacterales vs. Enterobacterales	93	20/32 vs. 20/61	0.0068	**0.0286**	3.42 (1.40–8.35)	Significant decrease
	*Pseudomonas* spp. vs. *Pseudomonas* spp.	79	0/9 vs. 9/70	0.2014	0.6850	0.34 (0.02–6.35)	Not conclusive
Tobramycin	Enterobacterales vs. Enterobacterales	72	5/32 vs. 6/40	0.9421	1.0000	1.05 (0.29–3.81)	No change
	*Pseudomonas* spp. vs. *Pseudomonas* spp.	78	1/9 vs. 13/69	0.5841	1.0000	0.54 (0.06–4.69)	Not significant
Amikacin	Enterobacterales vs. Enterobacterales	79	12/20 vs. 52/59	0.0076	**0.0475**	0.20 (0.06–0.67)	Significant improvement
	*Pseudomonas* spp. vs. *Pseudomonas* spp.	62	3/9 vs. 25/53	0.4510	0.6293	0.56 (0.13–2.48)	Not significant
Nitrofurantoin	Enterobacterales vs. Enterobacterales	72	21/32 vs. 8/40	0.0001	**0.0008**	7.64 (2.63–22.13)	Highly significant decrease
Fosfomycin	Enterobacterales vs. Enterobacterales	70	24/32 vs. 20/38	0.0512	0.1908	2.70 (0.97–7.51)	Borderline significance
Trimethoprim–sulfamethoxazole	Enterobacterales vs. Enterobacterales	94	1/32 vs. 9/62	0.1512	0.2519	0.19 (0.02–1.57)	Not significant
Colistin	Enterobacterales vs. Enterobacterales	61	20/20 vs. 36/41	0.0782	0.2519	6.18 (0.32–117.48)	Sustained high activity
	*Pseudomonas* spp. vs. *Pseudomonas* spp.	77	7/9 vs. 60/68	0.3951	0.4629	0.47 (0.08–2.65)	No relevant changes

OR: Odds Ratio; 95% CI: 95% Confidence Interval; FDR: False Discovery Rate (Benjamini–Hochberg adjustment for multiple testing). Notes: Statistical comparisons were performed using Pearson’s chi-square test or Fisher’s exact test, as appropriate. *p*-values were adjusted for multiple testing using the Benjamini–Hochberg FDR procedure. *p*-values < 0.05 after FDR correction are considered statistically significant and are highlighted in bold. Nitrofurantoin statistical comparison reflects overall Enterobacterales isolates tested via automated laboratory panels, evaluated using the *E. coli* breakpoint as an exploratory surrogate threshold. Note that official EUCAST clinical breakpoints are established exclusively for *E. coli* and are not recognized for non-*E. coli* Enterobacterales.

**Table 8:** Distribution of Gram-negative bacterial species according to metallo-β-lactamase (MBL) type and co-produced serine-carbapenemases (*n* = 182).

Bacterial Group/Species	Total Isolates (*n*)	VIM	NDM	IMP	NDM + IMP	MBL + OXA-48	MBL + KPC/MBL + KPC + OXA-48
Enterobacterales	94	21	14	15	5	37	2
*K. pneumoniae*	43	4	8	2	1	26 (NDM + OXA-48)	2 (1 VIM + KPC, 1 VIM + KPC + OXA-48)
*E. cloacae complex ^a^*	34	11	3	8	3	9 (7 NDM + OXA-48) (2 VIM + OXA-48)	0
*C. freundii*	7	3	1	1	0	2 (VIM + OXA-48)	0
*E. coli*	6	1	2	2	1	0	0
*K. oxytoca*	3	1	0	2	0	0	0
*R. ornithinolytica*	1	1	0	0	0	0	0
*Pseudomonas* spp.	86	86	0	0	0	0	0
*P. aeruginosa*	79	79	0	0	0	0	0
*P. putida*	7	7					0
*Aeromonas* spp.	2	0	0	2	0	0	0
*Aeromonas hydrophila/caviae*	2						0
*TOTAL*	182	107	14	17	5	37	2

^a^ Includes *E. cloacae* (*n* = 24), *E. hormaechei* (*n* = 6), *E. bugandensis* (*n* = 2), and *E. asburiae* (*n* = 2).

**Table 9:** *In vitro* susceptibility of aztreonam–avibactam and cefiderocol against Gram-negative isolates stratified by carbapenemase type and co-production (2021–2025 period).

Enzymatic Category/Mechanism	Aztreonam–Avibactam (*n*/*N*, % Susceptible)	Cefiderocol (*n*/*N*, % Susceptible)	Key Microbiological Remarks
Single MBL: VIM alone			
*Pseudomonas* spp.	35/45 (77.8%)	50/58 (86.2%)	Cefiderocol displays high activity against *P. aeruginosa* VIM
Enterobacterales	13/13 (100.0%)	12/13 (92.3%)	High activity maintained for both agents in Enterobacterales VIM+
Single MBL: NDM alone	7/7 (100.0%)	2/7 (28.6%)	Cefiderocol shows reduced activity against NDM producers
Single MBL: IMP alone	8/8 (100.0%)	4/7 (57.1%)	Aztreonam–avibactam retains 100% susceptibility
Dual MBL (NDM + IMP)	3/3 (100.0%)	1/3 (33.3%)	Aztreonam–avibactam susceptibility preserved
MBL + Serine-Carbapenemase Co-producers			
NDM + OXA-48	21/21 (100.0%)	2/21 (9.5%)	Avibactam inhibits OXA-48; Cefiderocol severely impaired
VIM + OXA-48	2/2 (100.0%)	0/2 (0.0%)	Avibactam inhibits OXA-48; Cefiderocol impaired
VIM + KPC/VIM + KPC + OXA-48	1/1 (100.0%)	0/1 (0.0%)	Avibactam inhibits KPC/OXA-48, restoring aztreonam activity

## Data Availability

No new data were created or analyzed in this study. Data sharing is not applicable to this article.

## Supplementary Materials

The following supporting information can be downloaded at: https://www.mdpi.com/article/10.3390/antibiotics15101010/s1, Supplementary Material S1: Methodological Quality and Risk of Bias Assessment.
