# Supplementary material for: In Vitro Activity of Aztreonam–Avibactam, Cefiderocol, and Conventional Antimicrobials Against Metallo-β-Lactamase-Producing Gram-Negative Urinary Isolates: A Systematic Review and Local Single-Center Retrospective Evaluation in a Tertiary Care Hospital (2016–2025) and Evaluation of Novel Agents (2021–2025)
Source: Antibiotics (Basel). 2026 Oct 11;15(10):1010. doi: 10.3390/antibiotics15101010 (PMC13700655; doi:10.3390/antibiotics15101010)
Supplement: Supplementary file 1 [file antibiotics-15-01010-s001.zip › antibiotics-4536864-supplementary.pdf]

Supplementary Material S1: Methodological Quality and Risk of Bias Assessment

To address the reviewer's request and guarantee the scientific transparency and validity of the synthesized findings, we conducted a systematic quality appraisal of the studies included in this systematic review. Systematic data extraction and methodological quality appraisal were conducted on the 22 fully accessible articles (references 17 through 38). Because these are preclinical, in vitro microbiological susceptibility investigations, standard clinical tools (such as Cochrane RoB or Newcastle-Ottawa) are not directly applicable. Therefore, we adapted the Joanna Briggs Institute (JBI) Critical Appraisal Tool for Prevalence Studies and focused on four critical methodological domains essential to minimize bias in clinical microbiology research:

Isolate Selection (Domain 1): Evaluates whether isolates were selected prospectively, consecutively, or represented a well-defined clinical cohort (Low Risk) vs. convenience or duplicate strains (High/Unclear Risk).

AST Standardization (Domain 2): Assesses whether susceptibility testing adhered to standardized reference methodologies (such as broth microdilution or agar dilution) and recognized consensus guidelines (Low Risk).

Breakpoint Criteria (Domain 3): Evaluates if interpretive breakpoints were explicitly sourced from up-to-date international reference committees (EUCAST or CLSI) (Low Risk) vs. qualitative synergy testing lacking formal clinical breakpoints (Moderate Risk).

Molecular Confirmation (Domain 4): Checks if the underlying carbapenemase genotypes (e.g., blaNDM, blaVIM, blaIMP) were confirmed using validated molecular techniques such as PCR, microarray, or whole-genome sequencing (Low Risk).

Study Reference	Isolate Selection (D1)	AST Method (D2)	Breakpoint Standard (D3)	Molecular Conf. (D4)	Overall Risk of Bias
[17] Kazmierczak (2016)	Low Risk	Low Risk	Low Risk	Low Risk	Low Risk
[18] Wenzler (2017)	Low Risk	Low Risk	Moderate Risk	Low Risk	Moderate Risk
[19] Falcone (2021)	Low Risk	Low Risk	Moderate Risk	Low Risk	Moderate Risk
[20] Zhang (2020)	Low Risk	Low Risk	Low Risk	Low Risk	Low Risk
[21] Cairns (2021)	Low Risk	Low Risk	Moderate Risk	Low Risk	Moderate Risk
[22] Bejarano (2022)	Low Risk	Low Risk	Moderate Risk	Low Risk	Moderate Risk

[23] Boattini (2023)	Low Risk	Low Risk	Low Risk	Low Risk	Low Risk
[24] Sempere (2022)	Low Risk	Low Risk	Moderate Risk	Low Risk	Moderate Risk
[25] Huang (2023)	Low Risk	Low Risk	Low Risk	Low Risk	Low Risk
[26] Falcone (2024)	Low Risk	Low Risk	Low Risk	Low Risk	Low Risk
[27] Freiberg (2024)	Low Risk	Low Risk	Low Risk	Low Risk	Low Risk
[28] Vega (2024)	Low Risk	Low Risk	Low Risk	Low Risk	Low Risk
[29] Wu Y (2024 - MS)	Low Risk	Low Risk	Moderate Risk	Low Risk	Moderate Risk
[30] Wu Y (2024 - IJAA)	Low Risk	Low Risk	Low Risk	Low Risk	Low Risk
[31] Blanco-Martín (2024)	Low Risk	Low Risk	Low Risk	Low Risk	Low Risk
[32] Klein (2025)	Low Risk	Low Risk	Low Risk	Low Risk	Low Risk
[33] Montero (2025)	Low Risk	Low Risk	Low Risk	Low Risk	Low Risk
[34] Pina-Sánchez (2025)	Low Risk	Low Risk	Low Risk	Low Risk	Low Risk
[35) Boattini (2025)	Low Risk	Low Risk	Low Risk	Low Risk	Low Risk
[36] Müller (2025)	Low Risk	Low Risk	Low Risk	Low Risk	Low Risk
[37] Rashid (2025)	Low Risk	Low Risk	Low Risk	Low Risk	Low Risk
[38] Sommer (2025)	Low Risk	Low Risk	Low Risk	Low Risk	Low Risk
